# Omega-3 Index and Cardiovascular Health

**DOI:** 10.3390/nu6020799

**Published:** 2014-02-21

**Authors:** Clemens von Schacky

**Affiliations:** 1 Preventive Cardiology, Medical Clinic and Poli-Clinic I, Ludwig Maximilians-University Munich, Ziemssenstr. 1, Munich 80336, Germany; E-Mail: Clemens.vonschacky@med.uni-muenchen.de; Tel.: +49-89-5506-3007; 2 Omegametrix, Am Klopferspitz 19, Martinsried 82152, Germany; E-Mail: c.vonschacky@omegametrix.eu

**Keywords:** cardiovascular disease, eicosapentaenoic acid, docosahexaenoic acid, omega-3 index, cardiovascular prevention

## Abstract

Recent large trials with eicosapentaenoic acid (EPA) and docosahexaenoic acid (DHA) in the cardiovascular field did not demonstrate a beneficial effect in terms of reductions of clinical endpoints like total mortality, sudden cardiac arrest or other major adverse cardiac events. Pertinent guidelines do not uniformly recommend EPA + DHA for cardiac patients. In contrast, in epidemiologic findings, higher blood levels of EPA + DHA were consistently associated with a lower risk for the endpoints mentioned. Because of low biological and analytical variability, a standardized analytical procedure, a large database and for other reasons, blood levels of EPA + DHA are frequently assessed in erythrocytes, using the HS-Omega-3 Index^®^ methodology. A low Omega-3 Index fulfills the current criteria for a novel cardiovascular risk factor. Neutral results of intervention trials can be explained by issues of bioavailability and trial design that surfaced after the trials were initiated. In the future, incorporating the Omega-3 Index into trial designs by recruiting participants with a low Omega-3 Index and treating them within a pre-specified target range (e.g., 8%–11%), will make more efficient trials possible and provide clearer answers to the questions asked than previously possible.

## 1. Introduction

Fish, marine oils, and their concentrates all serve as sources of the two marine omega-3 fatty acids eicosapentaenoic acid (EPA) and docosahexaenoic acid (DHA), as do some products from algae. To demonstrate an effect of EPA + DHA on heart health, a number of randomized, controlled intervention studies with clinical endpoints like overall mortality or a combination of adverse cardiac events were conducted in populations with elevated cardiovascular risk. One early intervention study with oily fish, rich in EPA + DHA, and some early studies with fish oil or fish oil concentrate or even purified EPA at doses ranging between 0.9 and 1.8 g/day indeed demonstrated effects in terms of fewer sudden cardiac deaths, fatal or non-fatal myocardial infarctions, or a combination of adverse cardiac events [1,2,3,4,5,6]. More recent trials did not demonstrate such effects [7,8,9,10,11,12]. Recent meta-analyses found no significant benefits on total mortality, cardiovascular mortality, and other adverse cardiac or cardiovascular events [13,14,15,16,17,18]. This is in contrast to findings in epidemiologic studies, where intake of EPA + DHA had been found to correlate generally with an up to 50% lower incidence of adverse cardiac events [18,19], and in even sharper contrast to epidemiologic studies based on levels of EPA + DHA, demonstrating e.g., a 10-fold lower incidence of sudden cardiac death associated with high levels of the fatty acids, as compared to low levels [20,21]. This seemingly contradictory evidence has led the American Heart Association to recommend “omega-3 fatty acids from fish or fish oil capsules (1 g/day) for cardiovascular disease risk reduction” for secondary prevention, whereas the European Society for Cardiology recommends “Fish at least twice a week, one of which to be oily fish”, but no supplements for cardiovascular prevention [22,23]. The more recent guidelines on treating patients with stable ischemic heart disease or patients after a myocardial infarction, targeting similar patient populations, do not recommend EPA + DHA [24,25]. At least in Europe, cardiologists do not routinely use EPA + DHA to reduce cardiovascular risk.

A similar picture emerges for atrial fibrillation: In epidemiologic studies, consumption of EPA + DHA or higher levels of EPA + DHA were associated with lower risk for developing atrial fibrillation, while intervention studies found no effect [26,27,28]. Pertinent guidelines do not mention EPA + DHA [29]. A similar picture also emerges for severe ventricular rhythm disturbances [20,21,30,31].

Why is it that trial results are at odds with results from epidemiology? What needs to be done to better translate the epidemiologic findings into trial results? The current review will try to shed some light on this issue, with a special consideration of the Omega-3 Index.

## 2. The Omega-3 Index as a Cardiovascular Risk Factor

At least some nutritional surveys do not provide valid data [32]. This may explain, why the relation of EPA + DHA in the diet to clinical events has been found to be looser than the relation of levels of EPA + DHA measured in blood to clinical events (e.g., [20,33]). A detailed discussion of the pros and cons of the various fatty acid compartments in which levels of omega-3 fatty acids (whole blood, whole plasma, plasma phospholipids, and others) should be measured is outside the scope of this review and can be found elsewhere [34]. The following points argue for the use of erythrocytes: erythrocyte fatty acid composition has a low biological variability, erythrocyte fat consists almost exclusively of phospholipids, erythrocyte fatty acid composition reflects tissue fatty acid composition, pre-analytical stability, and other points [34,35,36,37,38]. In 2004, EPA + DHA in erythrocyte fatty acids were defined as the Omega-3 Index and suggested as a risk factor for sudden cardiac death [39]. Integral to the definition was a specific and standardized analytical procedure, conforming the quality management routinely implemented in the field of clinical chemistry [39] In fatty acid analysis, methods have a large impact on results: when one sample was sent to five different laboratories offering determination of an Omega-3 Index, results differed by a factor of 3.5 [34]. While results may be internally valid in one laboratory, a difference by a factor of 3.5 makes it impossible to compare results among laboratories. Therefore, the Omega-3 Index was renamed HS-Omega-3 Index^®^. In contrast, the laboratories adhering to the HS-Omega-3 Index methodology perform regular proficiency testing, as mandated in routine Clinical Chemistry labs [34]. So far, the HS-Omega-3 Index is the only analytical procedure used in several laboratories. A standardized analytical procedure is a prerequisite to generate the data base necessary to transport a laboratory parameter from research into clinical routine. Moreover, standardization of the analytical procedure is the first important criterion for establishing a new biomarker for cardiovascular risk set forth by the American Heart Association and the US Preventive Services Task Force [40,41].

As exemplified by Table 1, the HS-Omega-3 Index has been measured in many populations. Of note, a lower HS-Omega-3 Index was always associated with a poorer clinical condition (Table 1).

**Table 1:** Mean HS-Omega-3 Index values in various populations, Mean (±standard deviation (SD)). Please note that in every population studied, a lower value was found to be associated with a worse condition than a higher value. References are given, if not, unpublished, *n =* number of individuals measured.

Population	HS-Omega-3 Index
**Western countries (high incidence of coronary heart disease)**	
*Germany*	
Unselected Individuals (*n* = 5000)	7.15 (±2.19)%
Patients with atherosclerosis [42], (*n* = 190)	5.94 (±1.41)%
Patients with hyperlipidemia [43], (*n* = 47)	7.00 (±1.90)%
Pregnant women, week 24 (*n* = 103)	7.66 (±1.83)%
Patients with congestive heart failure (*n* = 895)	3.47 (±1.20)%
Patients with major depression [44], (*n* = 90)	3.93 (±1.50)%
*Spain*	
Individuals with high risk for, but without cardiovascular disease [45], (*n* = 198) (SD not reported)	7.10%
*Norway*	
Patients with myocardial infarction [46] (SD not reported)	
With ventricular fibrillation (*n* = 10)	4.88%
Without ventricular fibrillation (*n* = 185)	6.08%
*Europe*	
Unselected data from routine determinations, *n* = 10,000	6.96 (+2.15)%
*USA*	
Healthy in Kansas City [47], (*n* = 163)	4.90 (±2.10)%
Framingham-Offspring [48], (*n* = 3196)	5.60 (±1.70)%
Patients with stable coronary heart disease [49], (*n* = 956) (SD not reported)	4.60%
Patients with major depression [50], (*n* = 118)	2.90 (±1.50)%
Adolescents with major depression [51], (*n* =150) (SD not reported)	3.46%
Patients with severe obstructive sleep apnea [52], (*n* = 52) (SD not reported)	4.00%
*Saudi Arabia*	
Individuals, most with diabetes (*n =* 69)	3.47 (±1.20) %
**Asian countries (low incidence of coronary heart disease)**	
*Korea*	
Healthy controls [53], (*n* = 50) (SD not reported)	11.81%
Healthy control [54], (*n* = 40)	10.55 (±0.48)%
Patients with myocardial infarction [53], (*n* = 50), (SD not reported)	9.57%
Patients with hemorrhagic brain infarction [54], (*n* = 40)	8.55 (±0.41)%
Patients with ischemic brain infarction [54], (*n* = 40)	8.19 (±0.64)%
Hemodialysis-patients without calcification on plain chest radiograph [55], (*n* = 11)	9.82 (±2.37)%
Hemodialysis-patients with calcification on plain chest radiograph [55], (*n* = 20)	9.23 (±2.34)%
Peritoneal Dialysis Patients [56], (*n* = 14)	12.83 (±3.30)%
Patients with a kidney transplant [57], (*n* = 49)	9.70 (±1.85)%
*Japan*	
Unselected men (*n* = 262), (SD not reported)	9.58%

All levels of fatty acids are determined by the balance of substance entering the body and those leaving the body. Neither a recent meal, even if rich in EPA + DHA, nor severe cardiac events altered the HS-Omega-3 Index [38,58,59,60,61]. However, while long-term intake of EPA + DHA, e.g., as assessed with food questionnaires, was the main predictor of the HS-Omega-3 Index, long-term intake explained only 12%–25% of its variability [46,62,63]. A hereditary component of 24% exists [64]. A number of other factors correlated positively (+) or negatively (−), like age (+), body mass index (−), socioeconomic status (+), smoking (−), but no other conventional cardiac risk factors [47,64,65,66,67,68,69,70,71]. More factors determining the level of the HS-Omega-3 Index, especially regarding efflux remain to be defined. Therefore, it is impossible to predict the HS-Omega-3 Index in an individual, as it is impossible to predict the increase in the HS-Omega-3 Index in an individual in response to a given dose of EPA + DHA [42,46,62,63]. In Table 2, current evidence is presented on the relation of the HS-Omega-3 Index to cardiovascular events.

This evidence is supported by measurements of EPA + DHA in other fatty acid compartments, as discussed in more detail elsewhere [72,73]. Within the framework of “Heart and Soul” and “Triumph”, it was investigated whether determination of the HS-Omega-3 Index added to the information obtained by assessing cardiovascular risk with a conventional scoring system, like the Framingham or GRACE scores for predicting fatal events. The HS-Omega-3 Index provided additional information, as demonstrated by larger areas under the curves in various c-statistics for fatal [74] and non-fatal events [53,75]. Taken together, the HS-Omega-3 Index predicts risk, appears largely independent of conventional risk factors, and adds to the information obtained by conventional risk scoring, thus fulfilling the second criterion for establishing a new biomarker for cardiovascular risk set forth by the American Heart Association and the US Preventive Services Task Force [40,41].

**Table 2:** Summary of epidemiologic studies relating the Omega-3 Index to cardiovascular events.

Acronym [reference]	Design	Disease	*n*	Years	Criterion	Comparison	Result
*Total mortality*							
Heart & Soul [49]	cohort	stable CAD	956	5.9	total mortality	HS-Omega-3 Index	HR 0.73; 95% CI, 0.56–0.94
Triumph [74]	cohort	recent MI	1144	2	total mortality	EPA in red cells tertiles	EPA < 0.25% total mortality 26%, 0.25 < EPA < 0.8% total mortality 13%, EPA > 0.80% total mortality 7%
Triumph [76]	cohort	recent MI	1424	1	total mortality	HS-Omega-3 Index < 4%*vs*. >4.0%	HR 2.0; 95% CI 1.2–3.3
Racs * [77]	cohort	recent ACS	460	2	total mortality	HS-Omega-3 Index in quartiles	not significant.
*Sudden cardiac death*							
[20]	case-control	SCD	82/108 cases/controls		SCD	red cell EPA + DHA in quartiles	OR 1.0–0.1 (95% CI 0.1–0.4)
Phys Health [21]	case-control	SCD	84/182 cases/controls		SCD	whole blood EPA + DHA in quartiles	OR 1.0–0.1 (95% CI 0.02–0.48) across quartiles
Cardiac morbidity							
[78]	case-control	ACS	94/94 cases/controls		ACS	whole blood EPA + DHA in quintiles	OR 1.0–0.2 (95% CI not reported), OR 0.67 (95% CI 0.46 to 0.98) per, 1 standard deviation increase EPA + DHA
[79]	case-control	ACS	768/768 cases/controls		ACS	HS-Omega-3 Index in tertiles	OR 1.0–0.31 (95% CI 0.14–0.67) across tertiles
[53]	case-control	ACS	50/50 cases/controls		ACS	HS-Omega-3 Index in tertiles	OR 1.0–0.08 (95% CI 0.02–0.38) across tertiles
no acronym [80]	case-control	ACS	24/68 cases/controls		STEMI	HS-Omega-3 Index in tertiles	OR 6.38 (95% CI 1.02–39.85)–1.0 across tertiles

Abbreviations: *n*: number of individuals studied; Coronary artery disease: CAD; HR: hazard ratio; MI: myocardial infarction; EPA: eicosapentaenoic acid; ACS: acute coronary syndrome; SCD: sudden cardiac death; DHA: docosahexaenoic acid; OR: odds ratio; STEMI: ST-elevation myocardial infarction. * No case estimate was reported in Racs. Therefore, by definition, it is unclear, whether the discriminatory power of the HS-Omega-3 Index was too small, or the study was too small to detect the discriminatory power.

Moreover, the HS-Omega-3 Index has made it possible to reclassify individuals from intermediate cardiovascular risk into the respective high risk and low risk strata [74,75], the third criterion for establishing a new biomarker for cardiovascular risk [40,41].

Increasing the HS-Omega-3 Index by increased intake of EPA + DHA in randomized controlled trials improved a number of surrogate parameters for cardiovascular risk: heart rate was reduced, heart rate variability was increased, blood pressure was reduced, platelet reactivity was reduced, triglycerides were reduced, large buoyant low-density lipoprotein (LDL)-particles were increased and small dense LDL-particles were reduced, large buoyant high-density lipoproteins (HDL)2 were increased, very low-density lipoprotein (VLDL1) + 2 was reduced, pro-inflammatory cytokines (e.g., tumor necrosis factor alpha, interleukin-1β, interleukins-6,8,10 and monocyte chemoattractant protein-1) were reduced, anti-inflammatory oxylipins were increased [43,81,82,83,84,85,86,87,88,89,90,91,92,93,94]. Importantly, in a two-year randomized double-blind angiographic intervention trial, increased erythrocyte EPA + DHA reduced progression and increased regression of coronary lesions, an intermediate parameter [95]. Taken together, increasing the HS-Omega-3 Index improved surrogate and intermediate parameters for cardiovascular events. A large intervention trial with clinical endpoints based on the HS-Omega-3 Index remains to be conducted. Therefore, the fourth criterion, proof of therapeutic consequence of determining the HS-Omega-3 Index, is only partially fulfilled [40,41].

## 3. Discussion of Neutral Results of Large Intervention Trials

Why is it that a low HS-Omega-3 Index can be a cardiovascular risk factor, and yet the results of the large trials testing EPA + DHA on clinical endpoints were neutral?

### 3.1. Bioavailability Issues

According to personal information from the respective first authors, participants of recent large intervention trials were advised to take their supplements, frequently an encapsulated EPA + DHA ethyl-ester with breakfast—in many countries a low-fat meal [7,8,9,10,11]. As discussed in more detail in a recent review, bioavailability of EPA + DHA depends on the chemical form in which they are bound (phospholipids > recombined triglycerides > triglycerides > free fatty acids > ethyl-esters) [96,97], on matrix effects (capsule ingestion with concomitant intake of food, fat content in food) or galenic form (*i*.*e*., microencapsulation, emulsification). The chemical binding form impacts on bioavailability roughly with a factor of two, whereas matrix effects can impact bioavailability up to a factor of 13, and the galenic form up to a factor of 21 [96,97,98,99]. When the large trials mentioned here were designed, the bioavailability issues just mentioned were unknown. Thus, involuntarily, the combination used in many of the large trials—An unemulsified ethyl-ester or triglyceride with a low fat meal—guaranteed a very low bioavailability of EPA + DHA.

### 3.2. Issues in Trial Design

In all large intervention trials conducted so far, study participants were recruited based on clinical conditions, but irrespective of their baseline omega-3 fatty acid status [1,2,3,4,5,6,7,8,9,10,11,12]. In all populations studied so far, the HS-Omega-3 Index had a statistically normal distribution (Table 1). Thus, the proportion of the study population with high levels was not prone to the effects of EPA + DHA, if any. In order to recruit a study population, in which an effect of EPA + DHA can be demonstrated, recruiting study participants with a low HS-Omega-3 Index is a logical choice.

In all large intervention trials conducted so far, study participants were exposed to a trial-specific, but fixed dose of EPA + DHA or placebo [1,2,3,4,5,6,7,8,9,10,11,12]. The inter-individual variability in response to a fixed dose of EPA + DHA has been found to be large, *i*.*e*., vary up to a factor of 13 [42,61]. This fact alone suggests individualizing the dose given in a trial, in order to reach a predefined target range of the HS-Omega-3 Index, e.g., 8%–11%. The statistically normal distribution of the baseline HS-Omega-3 Index further complicates this problem: A large overlap of omega-3 levels in the EPA + DHA group and placebo or control group can be expected, and has been seen in at least one large trial (Mühlhäusler, B., personal communication) [100]. With levels of omega-3 fatty acids not differing between intervention and placebo or control groups, a difference in study outcome cannot be expected, even if the condition studied would be susceptible to treatment with EPA + DHA. It is worth noting that when a neutral intervention trial was analyzed in a cross-sectional way, EPA + DHA levels directly related to study outcome and less to treatment allocation [101].

Conversely, if a trial reports a positive result, it is likely to have been conducted in a study population with low baseline levels of EPA and DHA, like congestive heart failure: a positive result of a large trial was reported [6], and we found a low mean HS-Omega-3 Index in patients with congestive heart failure (unpublished data, Table 1). A similar case can be made for major depression (Table 1, references [44,50,51,84]).

In the future, recruiting study participants with a low baseline HS-Omega-3 Index and treating them within a predefined target range will allow clearer trial results to be a distinct possibility. Dose adjustments will need to be performed in the placebo group. Since a larger treatment effect can be assumed in the study size estimation, it can be expected that study sizes will be smaller and thus studies less expensive. Clearly, these thoughts are not restricted to trials with patients with cardiovascular risk, atrial fibrillation or ventricular arrhythmia, but can be extended to all areas of omega-3 fatty acid research. This will facilitate scientific progress and lead to a faster recognition of the effects of EPA + DHA.

## 4. Conclusions

In an inconsistent manner, EPA and DHA are either recommended or not included in guidelines of cardiac scientific societies. The use of EPA and DHA is not supported by results of recent intervention trials or their meta-analyses. However, epidemiologic data based on assessments of diet and, even more so, data based on levels of EPA + DHA measured in humans, clearly demonstrate that EPA + DHA are associated with a low risk for total mortality, sudden cardiac arrest, and fatal and non-fatal myocardial infarctions. For a number of reasons, like a standardized analytical procedure and a large data base, levels of EPA + DHA are best assessed with the HS-Omega-3 Index. According to current criteria of the American Heart Association and others, the HS-Omega-3 Index is a novel cardiovascular risk factor. Moreover, the HS-Omega-3 Index has led to a fresh look at the field of omega-3 fatty acids and has made it possible to identify issues of bioavailability and study design, explaining at least in part the neutral results of previous intervention trials. In the future, more efficient intervention studies can be conducted based on the HS-Omega-3 Index, thus providing a clearer picture of the effects of EPA + DHA.
